# A Systematic Review on Nanoencapsulation Natural Antimicrobials in Foods: In Vitro versus In Situ Evaluation, Mechanisms of Action and Implications on Physical-Chemical Quality

**DOI:** 10.3390/ijms222112055

**Published:** 2021-11-08

**Authors:** Carini Aparecida Lelis, Anna Paula Azevedo de Carvalho, Carlos Adam Conte Junior

**Affiliations:** 1 Center for Food Analysis (NAL), Technological Development Support Laboratory (LADETEC), Federal University of Rio de Janeiro (UFRJ), Cidade Universitária, Rio de Janeiro 21941-598, Brazil; carinilelis@yahoo.com.br (C.A.L.); annacarvalho@iq.ufrj.br (A.P.A.d.C.); 2 Nanotechnology Network, Carlos Chagas Filho Research Support Foundation of the State of Rio de Janeiro (FAPERJ), Rio de Janeiro 20020-000, Brazil; 3 Laboratory of Advanced Analysis in Biochemistry and Molecular Biology (LAABBM), Department of Biochemistry, Federal University of Rio de Janeiro (UFRJ), Cidade Universitária, Rio de Janeiro 21941-909, Brazil; 4 Graduate Program in Food Science (PPGCAL), Institute of Chemistry (IQ), Federal University of Rio de Janeiro (UFRJ), Cidade Universitária, Rio de Janeiro 21941-909, Brazil; 5 Graduate Program in Chemistry (PGQu), Institute of Chemistry (IQ), Federal University of Rio de Janeiro (UFRJ), Cidade Universitária, Rio de Janeiro 21941-909, Brazil; 6 Graduate Program in Veterinary Hygiene (PPGHV), Faculty of Veterinary Medicine, Fluminense Federal University (UFF), Vital Brazil Filho, Niterói 24230-340, Brazil; 7 Graduate Program in Sanitary Surveillance (PPGVS), National Institute of Health Quality Control (INCQS), Oswaldo Cruz Foundation (FIOCRUZ), Rio de Janeiro 21040-900, Brazil; 8 Department of Biochemistry, Federal University of Rio de Janeiro (UFRJ), Cidade Universitária, Rio de Janeiro 21941-901, Brazil

**Keywords:** nanostructure, essential oil, food shelf life, food matrix x laboratory means, nanoemulsion

## Abstract

Natural antimicrobials (NA) have stood out in the last decade due to the growing demand for reducing chemical preservatives in food. Once solubility, stability, and changes in sensory attributes could limit their applications in foods, several studies were published suggesting micro-/nanoencapsulation to overcome such challenges. Thus, for our systematic review the Science Direct, Web of Science, Scopus, and Pub Med databases were chosen to recover papers published from 2010 to 2020. After reviewing all titles/abstracts and keywords for the full-text papers, key data were extracted and synthesized. The systematic review proposed to compare the antimicrobial efficacy between nanoencapsulated NA (nNA) and its free form in vitro and in situ studies, since although in vitro studies are often used in studies, they present characteristics and properties that are different from those found in foods; providing a comprehensive understanding of primary mechanisms of action of the nNA in foods; and analyzing the effects on quality parameters of foods. Essential oils and nanoemulsions (10.9–100 nm) have received significant attention and showed higher antimicrobial efficacy without sensory impairments compared to free NA. Regarding nNA mechanisms: (i) nanoencapsulation provides a slow-prolonged release to promote antimicrobial action over time, and (ii) prevents interactions with food constituents that in turn impair antimicrobial action. Besides in vitro antifungal and antibacterial, nNA also demonstrated antioxidant activity—potential to shelf life extension in food. However, of the studies involving nanoencapsulated natural antimicrobials used in this review, little attention was placed on proximate composition, sensory, and rheological evaluation. We encourage further in situ studies once data differ from in vitro assay, suggesting food matrix greatly influences NA mechanisms.

## 1. Introduction

For many years, one of the concerns of the food industry was microbiological contaminants, which can cause damage to consumers’ health. Foodborne diseases can be related to the ingestion of pathogens or their toxins. These diseases involve public health issues and economic loss for the industry [1]. In addition to the processing steps that aim to reduce microbiological contaminants, chemical additives also inhibit natural microbiota growth, preventing food deterioration. However, consumers increasingly concerned with health are looking for fresher and more natural foods [2]. Food innovation has emerged to offer compounds obtained from natural sources to replace the chemical additives used, increase the shelf life of products, improve sensory characteristics, and ensure fresher products and the closest-to-natural ones [3]. Very recently, reviewing several natural antimicrobials (NA) from different sources, in substitution to chemical additives, El-Saber Batiha et al. (2021) showed several adverse effects in foods, such as the impairment of sensorial properties [4].

Furthermore, these antimicrobials can interact with food components such as proteins, fats, sugars, and salts, decreasing their antimicrobial activity, requiring large amounts to inhibit microbial growth, compromising the sensory properties of foods such as taste, color, and odor [5]. Moreover, solubility and instability in temperature, light, and pH can compromise natural antimicrobials’ action [6,7,8,9,10]. To protect NA from these environmental conditions, its encapsulation has prospected in food preservation [4,11,12,13,14,15,16]. Microencapsulation of NA from different sources (e.g., plants, animals, microbial) was reviewed and shown to be a promising alternative to carrier various natural compounds and reduce microbial growth in different food products and food packaging, showing increased bioavailability, stability, targeted delivery, and facilitated controlled release [4,13,17].

Nanoencapsulation has been discussed with advantages over conventional microencapsulation due to the unique properties provided by the nanosized particle (1–100 nm) and its high surface/volume ratio, increasing the interaction with enzymes and microorganisms. However, there are still some challenges regarding the technology, and economic and regulatory concerns to its implementation in the food industry in relation to several factors, such as the influence of the wall material, the interaction with the food matrix, the behavior of the nanostructure during food processing, and the use of economic and straightforward strategies to obtain the nanostructures [18,19,20,21].

In the face of those challenges and the relevance of the subject to food preservation, an increasing number of research papers and literature reviews on the micro-/nanoencapsulation of natural bioactive compounds for several purposes in the food industry have been published in the last five years. Some studies are concerned with the characterization of nanostructures and nNA used in foods [22], the physicochemical properties and stability of nanoemulsions [23], gastrointestinal fate and bioavailability of nanoencapsulated food components [16]. Other approaches overview encapsulated NA (e.g., essential oils) incorporated in edible coating or active packaging materials in place of the food matrix [17,24,25]. Microencapsulation of natural antioxidants was recently reviewed to delay protein/lipid oxidation for meat preservation purposes [25]. Pateiro et al. (2021) reviewed nanoencapsulation of bioactive compounds, highlighting carbohydrates-, lipids-, and protein-based nanoparticles as carriers to design functional foods and nutraceuticals. Besides that, other authors also focus on the discussion of encapsulating techniques and systems reported to improve the efficiency of bioactive compounds at both micro or nanoscale, such as emulsion, spray-dryer, extrusion, freeze-drying, coacervation, liposomes, electrospray, and nanogels, among others [13,16,24,25,26]. Although many nanoencapsulated NA have been published and reviewed, most are produced on a laboratory scale. Only a few are on the market, mainly for economic and regulatory concerns. Besides that, a comprehensive view on quality and safety improvement of food products using nanoencapsulated NA, with delayed lipid oxidation and improved organoleptic properties, still lacks clarity [16].

Although several review articles focused on encapsulation techniques, materials characterization, or their application in food products, to the best of our knowledge no systematic review of the available literature was performed to compare the antimicrobial activity of free and nanoencapsulation form of NA into foods, addressing the differences between data of in vitro and in situ evaluation. The complex composition of foods (proteins, fats, carbohydrates, fibers, salts, minerals, and vitamins) differs from simpler laboratory methods. This difference may influence the types of interaction that nNA can perform, and consequently its antimicrobial action. Differences in composition result in a difference in the viscosity of the medium and are consequential in the diffusion processor. Thus, the accessibility of microorganisms for nNA in food may differ from the lab environment. From the articles related to safety improvements, we also review the effects on quality parameters of foods products (e.g., antioxidant activity, proximate composition, rheology, physical-chemical and sensory quality). Finally, we provide a comprehensive understanding of primary mechanisms of action of the NA nanoencapsulated into a food matrix. In this way, our work can address essential issues to help experts in the food industry improve the shelf life and quality of foods using nanoencapsulated natural compounds.

Although several review articles focused on encapsulation techniques, materials characterization, or their application in food products, to the best of our knowledge no systematic review of the available literature was performed to (i) compare data of in vitro and in situ evaluation of the antimicrobial activity of free and nanoencapsulation form of NA into foods; (ii) besides safety improvements, analyze the effects on the quality parameters of foods products rather than nutraceutical delivery (e.g., antioxidant activity, proximate composition, rheology, physical-chemical and sensory quality); and (iii) provide a comprehensive understanding of the primary mechanisms of action of the NA nanoencapsulated into a food matrix. In this way, our work can address essential issues to help experts in the food industry improve the shelf life and quality of foods by nanoencapsulated natural compounds.

## 2. Methodology

This review was carried out according to A. P. A. de Carvalho & Conte Junior (2020). The issue in this review was “Application of nanoencapsulated natural antimicrobials in food”. For this, we followed the recommendations of the Preferred Report items for Systematic Review and Meta-Analysis (PRISMA) (http://www.prisma-statement.org accessed on 21 September 2021). As support, we used a computational tool, State of the Art (Start) [27].

Based on population, intervention, comparison, and outcome (PICO), the research questions focused on: (i) which NA and strategies have been used to apply nanoencapsulation in foods? (ii) How far has it been in vitro from in situ evaluation methods? (iii) What are the main modes of action of nanoencapsulated NA in the food matrix? (iv) What is the influence on the physicochemical quality of foods?

To avoid possible sources of bias and based on the study’s objective, inclusion/exclusion criteria were used for eligibility.

SCREENING—Title, keywords, and abstract:

Inclusion: Studies published in English, at least one encapsulation strategy, at least one natural antimicrobial was encapsulated, applied in a food matrix.

Exclusion: Studies that are not research articles, no encapsulation method has been studied, no natural antimicrobials have been learned, they have not been applied to food, no antimicrobial activity has been inspected, application to packaging, films, sachets, coating materials, and for cleaning.

ELIGIBILITY—Full-text reading:

Exclusion: Natural antimicrobials and the encapsulation process were not in line with the objective of the study.

This research was carried out with the information acquired on 19 October 2020, in four electronic databases: Science Direct, Scopus, Web of Science, and PubMed. To search articles in the databases, we set up search strings, which were assembled based on keywords related to the research questions, synonyms related to encapsulation and antimicrobials, and the use of Boolean operators “AND”, “OR” and “NOT”. Search strategy: Science Direct: (encapsulation OR carrier OR delivery OR capsules) AND (“natural antimicrobial” OR “natural compost” OR “food preservation”) AND (food) NOT (“food packaging”); Scopus: (*encapsulation OR *carrier OR *delivery OR *capsules OR *structures) AND (“natural antimicrobial” OR “natural compound” OR “food preservation” OR “natural antibacterial” OR “natural antiviral” OR “natural antifungal”) AND (food) AND NOT (“food packaging”); Web of Science: (*encapsulation OR *carrier OR *delivery OR *capsules OR *structures) AND (“natural antimicrobial” OR “natural compound” OR “food preservation” OR “natural antibacterial” OR “natural antiviral” OR “natural antifungal”) AND (food) NOT (“food packaging”); PubMed: (encapsulation OR nanoencapsulation OR microencapsulation OR nanocarrier OR microcarrier OR carrier OR nanodelivery OR delivery OR microcapsule OR nanocapsule OR capsule OR microstructure OR nanostructure OR structure) AND (“natural antimicrobial” OR “natural compound” OR “food preservation” OR “natural antibacterial” OR “natural antiviral” OR “natural antifungal”) AND (food) NOT (“food packaging”). English was used for the research between 2010 and 2020 (based on previous analysis of encapsulated NA’s applied to food). Due to the objective of applying antimicrobials directly to food, studies with focus and discussion of NA encapsulated in packaging, films, sachets, or as food coating materials were excluded. Despite not having a single particle size that can be used as a definitive cut-off point to distinguish nanoparticles, such as nanoemulsions, from conventional particles, according to the literature, one of the points to be considered is that the average particle radius of the system must be less than 100 nm [28,29]. However, to avoid excluding possible works involving nanometric scale, but which were not emphasized in the text, the terms micro/nano or simply encapsulation were added.

After the selection process, the articles were imported into the Start^®^ tool. Duplicate/triplicate articles were automatically excluded, and the others went on to the stages of identification, selection, and extraction. Any doubts that arose during the articles’ identification were kept for further analysis based on the title, keywords, and abstract.

## 3. Results

### 3.1. Findings

**Figure 1:**
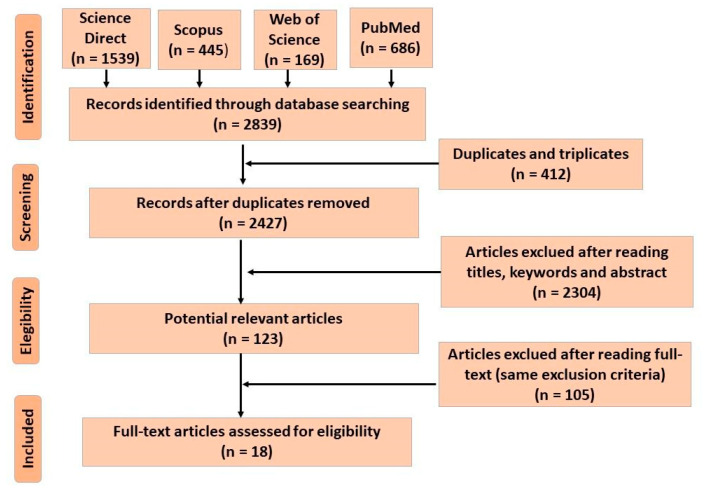
PRISMA flowchart-The results of the systematic search between 2010 and 2020.

The systematic review results were presented on the PRISMA flow chart illustrated in Figure 1, and Table 1 shows the list of selected articles in which nanoencapsulated NA studies were applied to food. Initially, 2839 articles were identified, of which 412 were duplicated/tripled, leaving 2427. After reading the title, keyword, and abstract, 2304 were excluded, and 123 were selected to read the full text. After careful reading, 105 articles were excluded for not meeting the eligibility criteria (size 1 at 100 nm, application in some food, not application in packaging), and 18 were selected. Other articles were added during the review discussion for further explanation of the subject.

### 3.2. Techniques Used for Nanoencapsulation of Natural Antimicrobials

As noted in Table 1, the technique that stood out in the process of nanoencapsulation of NA was emulsion.

The encapsulation by the emulsification method is based on the mixture of two immiscible and thermodynamically unstable liquids, one of which is dispersed in the other in the form of droplets. According to the nature of its dispersed phase, the emulsion is called water in oil (W/O), where the dispersed phase is water, and oil in water (O/W), in which the dispersed phase is oil. In the NA encapsulation analyzed in the studies, the emulsions used are O/W type, where the dispersed phase is oil. Most NA present hydrophobic characteristics and low water solubility, which justifies this type of emulsion (O/W). Formulating emulsions is thermodynamically unfavorable, requiring mechanical energy input or the supply of chemical energy obtained through low energy or high energy methods [48].

High energy methods use mechanical devices that generate intense, disruptive forces, causing a rupture and reducing the drops’ size. Thus, structures with reduced sizes, such as nanoemulsions, have unique properties and are more stable than emulsions. The high-intensity/frequency ultrasound method stands out in high-energy methods, which use sound waves to produce nanoemulsions. The technique made it possible to obtain emulsions with smaller diameters, high encapsulation efficiency (>80%), low polydispersity, low phase separation, and, consequently, high stability during storage [30,31]. In addition, it allows flexibility in the sonication time that influences the diameter of the droplets. Emulsions containing sesame oil had a smaller diameter with increasing sonication time (10, 20, and 30 min) (20 kHz; 750 W), reducing the diameter up to 80% with a 10 min increase in sonication time [32]. The longer the sonication time, the greater the total energy input to break the larger particles into smaller particles. The supply of mechanical energy can also be provided through high-pressure homogenization, which creates tiny drops, forcing high pressure through the passage of liquid through a valve with a narrow opening. The mixture can be passed through a high-pressure homogenizer repeatedly to obtain reduced sizes. Nanoemulsions of hexanal and trans-2-hexenal are stable for more than one year after being subjected to 14 passes at 300 Mpa [33]. When comparing pre-emulsions containing carvacrol obtained by high shear mixing and high-pressure homogenization, it was found that high-pressure homogenization demonstrated a better ability to form emulsions of smaller diameters (nanoemulsions), despite the emulsions obtained by the two methods showing stability over three months of storage at 4 °C [34,35]. Furthermore, although the emulsion obtained by high shear mixing had a larger diameter (174.8 nm) than high-pressure homogenization (74.4 nm), it caused a faster inactivation of *L. delbrueckii* and *S. cerevisiae*. This explains such an unexpected effect on the possible degradation of NA obtained by high-pressure homogenization [34]. Although the high energy method is significant in controlling particle size, zeta potential, emulsion turbidity, and encapsulation efficiency, possible degradation of NA is verified.

Associated with the effect of high energy methods used in nanostructures, such as nanoemulsions, the ratio of NA and surfactant also interferes with the size of the droplets [28,29,30,31,32,48].

However, low-energy methods for forming emulsions containing NA have gained popularity, as it is unnecessary to use specialized and expensive equipment. The procedures allow the production of tiny droplets without or with gentle agitation using only internal energy from the system. Due to minimal energy generation, the method has the advantage of avoiding the degradation of nanoencapsulated compounds [49].

Nevertheless, according to Chaudhari et al. (2021) the droplets formed by these methods are not stable at high temperatures. In this case, there is a need for a large amount of surfactant, in addition to being limited to a narrow range of compounds to be nanoencapsulated and surfactant type [50].

The formation of emulsions can occur due to the phase inversion temperature (PIT), where nonionic surfactants are typically used, in which their solubility is altered due to temperature changes. Thus, nonionic surfactants are highly hydrated at low temperatures, and the formation of O/W type emulsions is favored. However, by increasing the temperature, the shape of W/O type emulsions is preferred. At a phase inversion temperature, the droplets exhibit a very low interfacial tension, and the mean spontaneous curvature of the surfactant molecules is zero, promoting emulsification. However, the formation of tiny droplets and consequently high curvature makes the emulsions at this temperature unstable, with a high coalescence rate. Therefore, a rapid departure from the phase inversion temperature is necessary to obtain, for example, kinetically stable nano-emulsions. If the temperature deviation is fast heating, W/O emulsions will be accepted, while the temperature deviation by cooling allows obtaining O/W emulsions. Changing the W/O to O/W emulsion enables the encapsulation of the NA. The amount of material encapsulated significantly influences the average diameter and polydispersity of the formed nano-emulsions [36]. For the phase inversion composition (PIC) method, the emulsion formation occurs due to changing the system’s W/O ratio, keeping the temperature constant. Initially, it prepares an O/W type emulsion.

The subsequent addition of water leads to a progressive increase in the surfactant’s hydration degree, promoting a change in the surfactant’s spontaneous curvature. This method made it possible to produce nanoemulsions containing orange essential oils (EO) with stability for at least three months under refrigeration [37]. If we compare, the number of selected articles that used the high-energy method was more significant than the low-energy method to NA nanoencapsulation. This can be explained by the fact that the low-energy process has only gained popularity in recent years. By restricting our analysis to particles < 100 nm in size, the method may not have been able to form the nanostructures. More studies aiming at applying this method are necessary to reach the ideal conditions of encapsulation and thus obtain particles in nanometric scales.

Another method that uses chemical energy to promote the encapsulation process by forming emulsions is self-emulsification. In self-emulsification, there is no change in the surfactant’s spontaneous curvature. As shown by Stratakos & Grant (2018), it occurs due to the dilution process in which carvacrol and thyme oil was encapsulated using triglyceride oil and tween 80 [38]. Already Zhang & Zhong (2020) encapsulated thyme EO by self-emulsification, based on the group’s deprotonation present in the thyme, in aqueous alkaline conditions, followed by neutralization in the presence of sodium caseinate. Excellent emulsifying properties with EE greater than 90% were obtained [30]. The surfactant concentration has also been shown to influence the encapsulation process by this method. A higher concentration of surfactant (tween 80) allowed smaller, uniform, and stable droplets [39].

Emulsions are also used to encapsulate gel structures containing NA. Nanoemulsions containing gel structures formed by nisin and D-limonene have shown excellent stability stored at 28 °C for no less than three months, with no phase separation [40].

As can be seen, emulsification technology is the most common for the nanoencapsulation of NA. However, phenomena such as flocculation, sedimentation, coalescence, phase separation, cremation, and Ostwald ripening are the main challenges related to the stability of emulsions [51].

For better storage, emulsions can be subjected to additional processes such as lyophilization and drying [41].

Liposomes, spherical structures, are based on hydrophobic-hydrophilic interactions between phospholipid compounds and hydrophilic agents, allowing the formation of lipid vesicles or bilayers capable of transporting hydrophobic and hydrophilic NA. Despite the ability to spontaneously form bilayers in aqueous solutions, one of the most used methods for producing liposomes is the thin film method. This method consists of evaporating an organic solution containing surfactant and NA and an aqueous phase and hydrophilic material, followed by the entry of a sufficient amount of thermal energy. Thus, liposomes create physical barriers due to their amphiphilic nature, protecting natural antimicrobials from external conditions [42,52,53]. The particles formed may have their sizes reduced after being subjected to sonication, homogenized in cell ultra-fine grinder, among others. Then they are to be lyophilized or filtered for storage. The size of liposomes is influenced by factors such as type of antimicrobial, a ratio of natural antimicrobials and the membrane material used, temperature, and antimicrobial concentration [54,55].

Nanoliposomes of soybean and phosphatidylcholine (PC) containing lysozyme and nisin were coated with pectin and polygalacturonic acid. Although the coating does not differ significantly in size and polydispersity, the layer made the liposomes more negatively charged, providing greater electrostatic repulsion between the nanoparticles. Lysozyme’s nanoencapsulation efficiency (EE) decreased with the coating, mainly for pectin, which may be due to a rearrangement in the liposome after coating, releasing some lysozyme. The nanoliposomes encapsulating lysozyme-nisin did not differ from those without coating [42]. One of the limiting factors in applying liposomes is the low physical and chemical stability [56].

Another series of natural antimicrobials such as monoterpene, limonene, menthol, linalool, and thymol were nanoencapsulated using dripping ionic gelation. The authors were able to produce particles with diameters smaller than 55 nm. Despite the small size that helps in the stability of nanoemulsions, low values (−0.1690 mV) in the module of the zeta potential were observed [43]. We believe that a stability analysis during storage is of great relevance since low values in the module of the zeta potential represent low electrostatic repulsion among the nanoemulsions, which can reduce their stability.

Other methods for NA encapsulation are available in the literature, such as (i) a spray dryer consisting of solubilizing, homogenizing, and spraying the NA’s material in the drying chamber, in a stream of hot water air, instantly producing powder particles. The conversion occurs due to heat transfer from the air to the atomized droplets and the mass transfer of the atomized droplets to the air. Therefore, due to the rapid evaporation of the solvent, the natural antimicrobial is trapped. The starting material that feeds the sprayer can be a solution, emulsion, or suspension [57,58]. Despite having a good EE and industrial scaling, the use of high temperatures can damage sensitive compounds; (ii) precipitation that first involves the dispersion of the wall material and the active compound forming the solvent phase, followed by adding the solution dropwise in water with surfactant [59]; (iii) the complexation based on different interactions between two compounds, forming complexes that can be soluble or not; the coacervation that has involved the separation of two phases and occurs due to changes in pH, ionic strength, temperature, and the polymers’ structural characteristics. After phase separation comes a phase with high colloid concentration and the other dilution phase containing small colloid amounts. Polyelectrolyte complex and complex coacervation usually occur when electrostatic interactions are formed between molecules of opposite charge in an aqueous medium, thus decreasing the system’s free energy [60]. Despite the high load capacity, use of low temperatures, simple setup conditions, and no specific equipment, the complexity of the technique and high cost of the particle isolation procedure must be taken into account; (iv) the NA inclusion method, which involves cyclodextrins as a wall material, is considered a ready material. Cyclodextrins are cyclic oligosaccharides with a conical shape and hydrophilic outer wall, being soluble in water. The internal cavity is relatively hydrophobic due to the orientation of the glycosidic bonds. In this way, they can form inclusion complexes, total or partial, with hydrophobic molecules [61]; (v) electrospinning involves the action of an external electric field in which continuous fibers with high porosity and surface/volume ratio are formed [62,63]; (vi) methods such as freeze-drying and microfluidics are also used, although they are not very recurrent. The encapsulation for freeze-drying consists of dissolving, dispersing, or emulsifying the NA in the wall material followed by lyophilization. The lyophilization process can generate many stresses for the material to be encapsulated due to the freezing and dehydration process. The molecules used as the wall material must present a certain degree of cryoprotection, helping stabilize the active material [64,65].

Despite the potential these techniques demonstrated in encapsulating NA, they could not produce structures with a diameter < 100 nm, which is the focus of our review [66,67,68,69]. Most of these articles were presented in other reviews available for reading.

Through the data obtained from the articles, it is not possible to determine which is the best method to obtain structures with a diameter < 100 nm, since factors such as the type of wall material, the ratio surfactant/NA, and the oil used (in the case of emulsions) demonstrate the influence of the size of the formed structure. However, the formation of nanoemulsions stood out the most for the nanoencapsulation of NA.

We believe that studies aimed at the development of new encapsulation methods and analyses that propose novel wall materials, studies trying to improve existing processes, the best conditions for the formation of nanostructures, and the choice of the best wall material for each technique is interesting in order to achieve industrial application faster and more efficiently.

### 3.3. In Vitro Efficacy of Nanoencapsulated Natural Antimicrobials

When analyzing Table 2, we verified that the main NA submitted to studies of nanoencapsulation and food application is obtained from plant sources such as EO and extracts and microbial sources such as bacteriocins, in addition to lysozyme.

The minimum inhibitory concentration (MIC) for *Escherichia coli* (*E. coli*) was changed when the NA was encapsulated. In addition, differences were observed among studies. Limonene, linalool, menthol, and thymol have been shown to reduce MIC from 48% to 72% in nanoencapsulated form, compared to free NA. A mixture of terpenes (composed mainly of terpinen-4-ol, p-cymene, thujol, cyclohexanol (4-isopropyl-1-methyl)-trans, cyclohexanol, terpinolene, and δ-terpinene) reduced the MIC for *E. coli*. It showed without significant effect for the minimum bactericidal concentration (MBC). Thymol also confirmed the MIC reduction by 20%, and thyme reduced by 50%. As observed for MIC, thyme EO was more effective in lowering MBC (66.6%) for *E. coli* after nanoencapsulation compared to the effect of nanoencapsulation for thymol (20%) [30,34,43,44]. Thyme essential oil is composed of several molecules emphasizing thymol and carvacrol, both with antimicrobial activity [70,71]. We suggest that the more significant effect of thyme essential oil on the *E. coli* activity may be associated with the synergistic effect between the components (mainly thymol and carvacrol) present in the essential oil, different from the isolated form (only thymol) [72,73]. Furthermore, it is crucial to mention that the wall material used in the nanoencapsulation process is different, influencing the nanostructures’ antimicrobial activity. The pH has been shown to affect the effectiveness of nanoencapsulated sweet orange EO on *E. coli*. At pH 4 the nanoencapsulated sweet orange EO reduced by 66% *E. coli* compared to free form. However, at pH 7 this effect was not observed [37]. We suggest that the low effectiveness of sweet orange EO at pH 7 may be associated with the low solubility of chitosan, used as a wall material to form nanoemulsions [74]. Hexanal increased the MIC and MBC by 150% and 50%, respectively, and trans 2-hexanal increased the MIC and MBC by 40% [33]. Therefore, the nanoencapsulation process does not continually improve the antimicrobial effect of NA. We suggest that the diffusion of the hexanal and trans 2-hexanal of the nanoemulsion was slow enough to not demonstrate its antimicrobial potential over the study period, which may allow microbial growth at the beginning of study [31]. The MIC of limonene, linalool, menthol, and thymol for *Salmonella* Typhimurium was 30% to 72% lower in the nanoencapsulated form than in the free form [43]. However, nanoencapsulated linalool demonstrated a 72% reduction in the MIC of *S.* Typhimurium in the study by Badawy et al. (2020) [43]. Prakash et al. (2019) showed a decrease of only 0.625% in the MIC compared to the free form, demonstrating that possibly the wall material significantly influenced the diffusion of the natural antimicrobial to the external environment and its antimicrobial effect [31]. In addition, data regarding the encapsulation efficiency would be interesting since the determined MIC is from the nanoencapsulated system, and the amount of NA present in the nanoemulsion/nanoparticle may be different due to the encapsulation efficiency. Despite the low effect for the MIC of linalool nanoemulsion, compared to free form, the nanoemulsion demonstrated an 11.5% greater inhibition of biofilm formation [31]. More promising results were found for nanoemulsions containing trans-cinnamic acid that reduced the MIC of *S*. Typhimurium by 87.5% [39].

Nanoemulsions containing 0.003%, 0.03%, 0.3% eugenol have been shown to reduce *Staphylococcus aureus* from 50% to 100% compared to free eugenol [32]. After being nanoemulsified in trans-cinnamic acid, the MIC and MBC for *S. aureus* were decreased by 87%, in a concentration of 0.78 mg/mL (MIC) and 3.13 mg/mL (MBC) [39]. In this case, the expressed concentration is that of the nanoemulsion containing the trans-cinnamic acid. In the study by Ghosh et al. (2014), the concentration of eugenol present in the nanoemulsion was expressed [32]. Nanoemulsified oregano EO reduced the growth of *S. aureus* by up to 40% in a 5% concentration of EO oregano in the nanoemulsion [36]. When comparing this data with that obtained by Ghosh et al. (2014), we found that the nanoemulsified oregano EO was less effective in reducing the growth of *S. aureus* than nanoemulsified eugenol. This can be explained by the different wall materials and oils used in the NA nanoencapsulation process and the different sizes of the nanoemulsions obtained. The smaller size of the nanoemulsion, the greater the area of contact with the external environment. Intermolecular interactions between the core material (NA) and the wall material can occur during nanoencapsulation. These interactions may have different magnitudes depending on the other molecules used, influencing NA diffusion from the nanoemulsion to the external environment. The wall material will also influence the solubility and stability of the hydrophobic compounds, directly reflecting on their antimicrobial activity. A 0.2% concentration of nanoemulsion containing thyme EO reduced the MIC and MBC for *S. aureus* by 33.3% compared to free thyme EO, supporting those above [30]. Contradictorily, hexanal increased by up to 100% the MIC and without effect for MBC for *S. aureus*. Trans 2-hexanal increased the MIC and MBC for *S. aureus* by up to 150%, and both compared to their free forms [33]. In this case, studies with other wall materials and other nanoencapsulation methods may be engaging in efforts to improve the effect of hexanal and trans 2-hexanal as NA.

The results found for *Listeria monocytogenes* were not so promising. Nanoemulsion containing thymol showed less effect for MIC than free thymol and increased MBC by 14.2% after nanoencapsulation [44]. The MIC of hexanal rose from 25% to 200%, and MBC increased up to 150% after nanoencapsulation. Trans 2-hexenal increased MIC and MBC up to 60% after nanoencapsulation. The results found were dependent on the incubation time and the initial concentration of the tested microorganism. In general, the MIC was higher after 48 h than 24 h and higher in higher concentrations (CFU/mL) of the inoculated microorganism (*L. monocytogenes*) [33]. Malheiros et al. (2016) [45], when using liposomes containing bacteriocins, obtained a reduction of 2% in the log CFU/mL for *L. monocytogenes* at 30 °C. However, at 7 °C, it had no effect compared to free bacteriocins. We believe that at a temperature of 30 °C, more significant agitation of the molecules, compared to 7 °C, facilitated the diffusion of bacteriocin outside the nanostructure and in the food, making it more effective as an antimicrobial. *L. monocytogenes* is a pathogenic microorganism frequently associated with food contamination, representing risks to consumer health, making new studies with nNA highly relevant for its inhibition.

The activity of trans-cinnamic acid against *Pseudomonas aeuroginosa* was also improved after nanoencapsulation, reducing the MIC and MBC by 50% compared to the free form [39]. Nanoemulsified mixture terpenes reduced the MIC by 90% and the MBC by 50% to *Saccharomyces cerevisiae* [34]. Nanoemulsions of hexanal and trans-2-hexenal, after 24 h of incubation and with an initial microorganism count of 6 log CFU/mL, reduced the MIC by 20% and 66.6% for *S. cerevisiae* compared to its free forms, showing that the mixture of nanoencapsulated terpenes was more promising against *S. cerevisiae* than hexanal and trans-2-hexenal nanoencapsulated [33,34].

When comparing the effects of the same nanoencapsulated NA against *E. coli*, Gram-negative microorganism, and *S. aureus*, Gram-positive microorganism, there was greater effectiveness of *Origanum majorana* EO and thyme EO against *E. coli* [30,36]. The opposite result was obtained by Bei et al. (2015) [36], in which the MIC of nanoemulsions containing nisin and D-limonene was higher for *E. coli* compared to *S. aureus,* demonstrating that, despite the Gram-negatives, microorganisms are more susceptible to the antimicrobial action of EO, and that this cannot be generalized and extrapolated to the nanoencapsulation process.

The association of NA can be used to improve antimicrobial activity, as demonstrated by Bei et al. (2015) [36], who found a synergistic effect between nisin and nanoencapsulated D-limonene compared to the action of nanoencapsulated antimicrobials alone.

Effects against fungi and their toxins were also evaluated. *Origanum majorana* EO nanoencapsulated more effectively against *Aspergillus flavus* (reduced the MIC by 60%) and aflatoxin B1 (AFB1) (reduced the MIC by 33.3%) compared to the free forms [41]. *Coriandrum sativum* EO reduced the MIC of a mixture of 14 fungi by 44.4% and the MIC for AFB1 by 50% compared to free forms [46], demonstrating that the nanoencapsulation process helps in the antimicrobial effect against fungi and their toxins.

Through the demonstrated results we can conclude that nanoencapsulation, in general, improves the effect of NA on several microorganisms. The nanoencapsulation process can enhance the solubility of NA in aqueous media and strengthen its stability in temperature, pH, and light, which provides improvements for application. However, it is essential to emphasize that when considering nanoencapsulation of the NA, it must be taken into account that the NA must cross the protective barrier to act on the microorganisms, which can hinder its immediate action. Possible intermolecular interactions between NA and the materials used to form the nanostructures will also make it difficult for the NA to exit to act on microorganisms.

### 3.4. In Situ Efficacy of Nanoencapsulated Natural Antimicrobials

The food matrix is complex and diverse, formed by water, proteins, lipids, carbohydrates, vitamins, fibers, and ions. In addition, these molecules are arranged in different forms, forming different structures. The molecules can be soluble, dispersed, forming clots, structured networks (gels), and interacting with each other and with other molecules. These characteristics make food different from the culture media used in laboratories. Therefore, in situ studies (food) are relevant to an accurate explanation of the nNA. Table 3 shows the effects of NA in situ (food) studies.

Trans-cinnamic acid, when nanoencapsulated, demonstrated effectiveness against aerobic mesophilic bacteria and aerobic psychrophilic bacteria present in fresh-cut lettuce, reducing 50% of the growth of the microorganism compared to free antimicrobial [39]. Linalool, after nanoencapsulation, demonstrated greater effectiveness in inhibiting biofilms produced by *S*. *Typhimurium* in fresh-cut pineapple [75], and 1 log cycle of *E. coli* in zucchini was reduced after nanoemulsification of carvacrol [35]. We found that despite few studies involving the application of nNA in fruits and vegetables, the results are promising, indicating greater effectiveness of nNA.

Donsì et al. (2014) [35] also analyzed carvacrol nanoemulsions in cooked meat sausage and demonstrated greater effectiveness (reduction of 1.5 log cycles) against endogenous microbial population than its free form. The total viable count was reduced by approximately 7% for cinnamon oil after nanoencapsulation. The reduction in microbial growth was dependent on the concentration used, but it was not proportional. The 8-fold increase in concentration reduced 0.83% more microbial growth [47]. The same effect of attention was observed for Badawy et al. (2020) [43], where the increase in concentration increased the inhibitory effect for *E. coli*. However, this effect was not proportional. Limonene, menthol, linalool, and thymol nanoencapsulated reduced the growth of *E. coli* in minced meat by approximately 76%, 66%, 72%, and 75%, respectively, compared to free form. The 2.5-fold increase in concentration reduced 3%, 6%, 14%, and 9% more *E. coli* growth [43]. A reduction of 15% and 11% in the evolution of *E. coli* and *S. aureus* was obtained using oregano EO nanoencapsulated in chicken pate. However, in this study, the control was chicken pate without the addition of antimicrobial [36]. Both carvacrol and thyme oil, when nanoencapsulated, reduced the growth of *E. coli* in beef by 20% and 30%, respectively, compared to free forms [38]. These studies have shown that meat products have been susceptible to the antimicrobial action of nNA, showing promising results.

At 2.5 times lower concentration of oregano, EO (*Origanum majorana*), nanoencapsulated did not show any effect against aflatoxin B1 (AFB1) in maize compared to its free form [41]. *Coriandrum sativum* EO nanoencapsulated also showed no impact on AFB1 in rice compared to its free form. However, it increased the protection against fungal infestation by 25.6%. When using 2xMIC (1.0 μL/mL), the effect was less (21.7%) than the MIC (0.5 μL/mL) (25.6%) [71], demonstrating once again that the increase in the concentration of nNA does not increase the antimicrobial effect proportionately and, in some cases, no growth is observed.

*Lactobacillus delbrueckii* in juice (orange and pear) was reduced by 37.5% using 1.0% of nanoencapsulated mixture terpenes. The reduction was total in 5% and 10% concentrations compared to the control, without antimicrobial [34]. Nanoencapsulated Eugenol (0.3%) demonstrated a better antimicrobial effect, 15% reduction against bacteria in orange juice when compared to sodium benzoate (0.3%), a chemical preservative widely used in the food industry [32]. For *S. aureus*, the trans-2-hexanal and hexanal mixture after nanoencapsulation reduced 1 log cycle on the 2nd day and 1.5 log cycles on the 8th day at 10 °C. For *L. monocytogenes* and *E. coli*, there was an increase of 1 log cycle and 0.5 log cycle on the 2nd day, respectively, and a reduction of 1 log cycle and 0.5 log cycles on the 8th day, at 10 °C. For *S. cerevisiae*, a decrease of 2 log cycles was observed on the 14th day, and on the 22nd day showed without effect, both at 10 °C [33]. Nanoencapsulated thymol demonstrated great effectiveness against *L. monocytogenes* and *E. coli* in cantaloupe juice, below the detection limit in 2 h, compared to the free form that presented 5 log cycles [44]. No effect of sweet orange EO nanoencapsulated, when compared with free, was observed in juice (orange and apple) against *E. coli* during 30 min of exposure [37].

The effectiveness of unencapsulated thymol varied according to the fat content present in the milk. Comparing the full-fat milk and 2% reduced-fat milk that used the same concentration of the nanoemulsion containing thymol (4.5 g/L), greater effectiveness was observed in the 2% reduced-fat milk. The effect against *L. monocytogenes* was close (~20%) for skim milk and full-fat milk. The concentration used in full-fat milk was 4.5 times higher. Greater effectiveness against *E. coli* was observed in full-fat milk (72.2%) compared to skim milk (47.3%). However, the concentration used in full-fat milk was higher (4.5 times). Regardless of the difference observed as a function of the fat content in milk, in both situations nNA was more effective than its free form against *E. coli* and *L. monocytogenes* [44]. The influence of fat percentage on antimicrobial effectiveness was also demonstrated by Lopes et al. (2019) [42]. However, the results obtained were contradictory to that of Xue et al. (2017) [44]. In the study by Lopes et al. (2019) [42], the effect of nisin and lysozyme nanoencapsulated compared to free form on the growth of *L. monocytogenes* was more significant in whole milk than in skim milk, both at 37 °C and 7 °C of storage. In skim milk, an increase of up to 3.5 log cycles was observed over 10 h. Nisin and lysozyme nanoencapsulated showed no effect on *S. enteritidis* and mixtures of *Listeria* strains in milk (whole milk and skim milk) at 37 °C and 7 °C. No effect was observed against *L. monocytogenes* in UHT goat milk at 37 °C and 7 °C of storage when bacteriocins were nanoencapsulated [72]. The total microbial counts were reduced by up to 80% when D-limonene and nisin were nanoencapsulated. In this case, the control was milk without antimicrobials [40]. Thymol nanoencapsulation reduced MIC and MBC by 12.5% for *E. coli*, 50% by MIC, and 35.5% by MBC for *S. aureus* in milk, compared to free NA [30].

We can verify that nanoencapsulation makes NA more promising to be applied in food. The effect of nNAs is variable depending on the different types of food and possible variations in terms of composition that they may present. Therefore, further studies of nNA in food matrices are necessary to understand better and clarify its effect.

### 3.5. Comparison between In Vitro and In Situ Efficacy

Some studies have demonstrated greater effectiveness in minced meat and milk. However, a higher concentration of nanoencapsulated antimicrobials was used in the food than in the laboratory environment [30,40,43,44]. For UHT goat milk added with nanoencapsulated bacteriocins at a temperature of 30 °C of storage, a slight difference was observed for the in vitro study, being more effective in the in vitro study reduction of *L. monocytogenes*. As for the storage temperature of 7 °C, no difference was observed in the in vitro study of the UHT goat milk [45]. Chaudhari et al. (2020) demonstrated no difference between the in vitro and maize studies regarding the effect of nanoencapsulated oregano EO against AFB1. Nanoemulsion of sweet orange EO showed a negligible effect on juice against *E. coli* [37]. The nanoencapsulated *Coriandrum sativum* EO was less effective in protecting against fungal infestation and AFB1 in rice than in vitro study [46]. Trans-cinnamic acid nanoencapsulated also had a lesser effect against aerobic bacteria and aerobic psychrophilic bacteria in fresh-cut lettuce than the in vitro study against *S. aureus*, *S. typhimurium*, and *P. aeuroginosa* [39].

The laboratory methods used in microbial growth are simpler than food in terms of composition. Some foods are more complex than others in composition and structure, explaining the differences found in different foods. In addition to composition, differences in viscosity can influence the diffusion of the natural antimicrobial and its action. Therefore, it is important to study different foods since their composition and rheological characteristics vary, influencing different nNA. Although in vitro studies are very relevant, they cannot be extrapolated to food, demonstrating the importance of a more excellent linkage between NA research in vitro and in situ.

### 3.6. Antimicrobial Modes of Action of Nanoencapsulated NA in Foods

Of the articles selected in our studies, some analyzed the mechanisms of action of nNA. In these studies, the primary mechanism of action involves damage to the cell membrane with cell constituents and cell lysis release. Such damage to the cell membrane can be irreversible, and components such as proteins and nucleic acids can be released. In the case of fungi, damage to the plasma membrane also occurs, inhibiting the content of ergosterol, causing cell leakage of ions (Ca2^+^, Mg2^+^, and K), nucleic acids, and proteins, in addition to inhibiting methylglyoxal. A reduction in carbohydrate catabolism in the presence of EO is also one reason for the inhibitory action of mycotoxins’ occurrence [31,32,39,40,41,75]. Cell membrane damage and leakage of cell constituents have been confirmed in other studies using D-limonene and thymol, methyl, cinnamate, and encapsulated linalool [76,77,78].

For EO in general, the mechanisms of antimicrobial action are also associated with EO crossing through the cell cytoplasmic membrane and mitochondria, permeating their different layers of fatty acids, polysaccharides, and phospholipids, increasing the permeability of the cell [79,80]. This membrane disturbance has consequences such as reducing the membrane potential, the leakage of ions and cellular contents in general, reducing the ATP pool, and even the loss of macromolecules, leading to cell lysis [79,81]. Coagulation of the cytoplasm and enzyme inhibition, which influences energy regulation and the synthesis of structural components, has also been associated with the mechanism and action of EO [79,80].

Bacteriocins, such as nisin, also show more significant antimicrobial properties against Gram-positive bacteria than Gram-negative bacteria. The generally accepted model of action for nisin in cells involves the formation of pores in target cells’ cytoplasmic membrane, leading to the efflux of small essential cytoplasmic components, such as amino acids potassium ions and ATP, and the pH gradient of bacteria [82].

**Figure 2:**
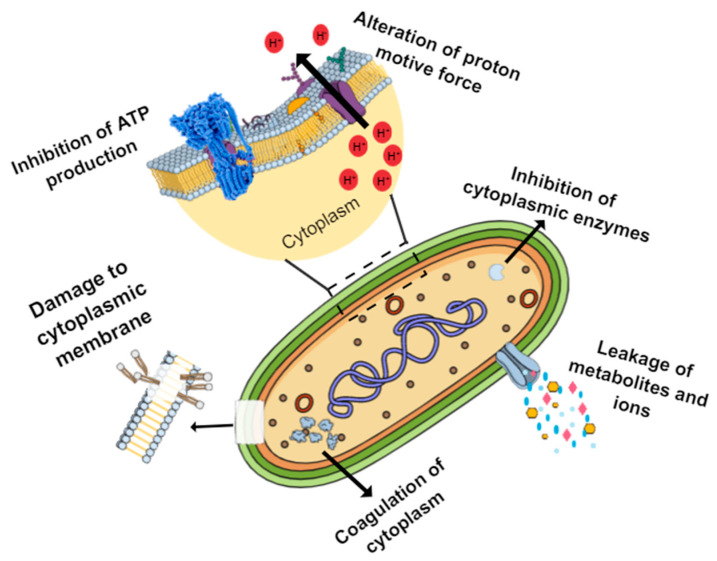
Principal targets of natural antimicrobials in bacterial cells and some mechanisms of antimicrobial activity.

The main mechanisms of action of natural antimicrobials are shown in Figure 2.

As we can see, the mechanism of natural antimicrobials is not unique, causing several effects on cells that are similar to those found when antimicrobials are nanoencapsulated. If the mechanisms are the same, why are the antimicrobial activity of natural antimicrobials in most studies improved after the nanoencapsulation process? In nNA, their release is slow and prolonged, which provides an antimicrobial effect over time [35,39,44,46,83]. Nanoencapsulation of natural antimicrobials prevents them from interacting with food constituents and ceasing to exercise their antimicrobial action [36]. Structures in reduced sizes (nano) may have a particular facility to pass through microbial cells and interfere with vital cell processes [31,33,37]. The wall material used in the formation of the nanostructure can interact with the surface of the microbial cell (interact by inter-membrane transfer, release contact, absorption, fusion, and phagocytosis) and form pores, which facilitates the action of the antimicrobial and at the same time increases the permeability of the cell, in addition to allowing continuous diffusion of antimicrobial compounds across the cell membrane [33,41,84]. In addition, nanomaterials, such as nanoemulsions, can easily permeate porous proteins in the bacterium’s outer membrane, facilitating NA delivery [85]. In bacteria, which are structures with a resulting negative charge, the charge resulting from the nanostructures formed will influence the process of electrostatic interaction with the bacterial membrane [43,86]. The nanoencapsulation process is related to the increase in NA solubility. The rise in polarity due to the NA coating reduces the interfacial tension, immiscibility in aqueous systems that represent most foods. This makes NA distribution in the food more accessible and evenly [40,43,44]. Nanoencapsulation also protects NAs from possible degradation due to the increase in temperature, the presence of light, oxygen, and moisture [37,40]. It is important to note that microbial inhibition by NA can be influenced by the environmental challenges of microorganisms, such as low temperatures, organic acid stress, and osmotic stress [42].

### 3.7. Antioxidant Effect of Nanoencapsulated NA

Natural antimicrobials can act as antioxidants, helping to preserve food. The antioxidant properties (Table 4) provided by NA are favorable in maintaining the oxidative stability and the characteristics of food.

Better antioxidant effect was observed for nanoemulsions containing *Coriandrum sativum* EO reducing by 36% in IC50 for DPPH• and 26% in IC50 for ABTS• + compared to free. The larger surface area of the nanostructures increases exposure to DPPH and ABTS radicals and reduces malondialdehyde production by 70% when nanoencapsulated [46]. Reduction of malondialdehyde (46%) was also observed after the nanoencapsulation of EO *Origanum majorana* [41]. Lipid oxidation based on the peroxide value was also analyzed. It was found that limonene, linalool, menthol, and thymol reduced peroxide values from 31% to 37.5% after the nanoencapsulation process [43]. At a concentration of 11429 mg/L nanoemulsions containing cinnamon oil, it was also able to reduce 2-thiobarbituric acid reactive substances (TBARS) by up to 44% compared to antimicrobial in free form [47]. After nanoencapsulation, limonene, linalool, menthol, and thymol increased antioxidant activity by 6.0% to 10.4% [43]. These data demonstrate that in addition to acting in the fight against microorganisms, NA can also act as natural antioxidants, and the improved effect after nanoencapsulation may be related to the slow and gradual release of NA, more excellent solubility in food, and possible stability of temperature, light, and oxygen.

### 3.8. Implications on Nanoencapsulated NA on Quality Parameters and Physical-Chemical Properties of Foods

The addition of natural antimicrobials can modify the characteristics and properties of foods. Composition, color, pH, Brix, rheological properties, and sensory properties of food were analyzed (Table 4). The changes in composition, sensory, physical-chemical, and rheological characteristics can make food unfeasible in legislation and consumer acceptance.

Nanoemulsion of oregano EO has been shown not to alter the composition of chicken pate [47], while nanoemulsion containing limonene, linalool, menthol, and thymol, despite not changing the content moisture and total protein of minced meat, has been shown to alter the fat content, this alteration being dependent on the NA and the concentrations used. A slight increase in ash content of 0.02% to 0.10% was observed when a concentration of 2500 mg/kg of nanoemulsions containing NA was used [43].

The degradation of sea bass fillets by microorganisms may be associated with the degradation of proteins and non-protein nitrogenous compounds and, consequently, the increase of total volatile basic nitrogen (TVB-N). However, when applying cinnamon oil, it has been shown to reduce the TVB-N formation, being that the nanoencapsulated form decreased the TVB-N from 2.5% to 15% compared to the free form [47]. These data are probably related to the reduction in the total viable count observed in the microbiological analysis.

The results so far are promising, showing that natural antimicrobials can also increase antioxidant activity in food and act on food preservation. However, sensory and rheological characteristics are also essential to be analyzed so that the insertion of natural antimicrobials does not reduce the acceptance of food by the consumer. Orange juice added with sweet orange EO nanoemulsions showed a 14% reduction on the hedonic scale than the control (juice without natural antimicrobial). No significant difference was observed for apple juice added from the same nanoemulsion [37]. However, fresh-cut pineapple added with nanoemulsions containing linalool EO had an increase of 50%, 20%, 14%, and 40% in appearance, pineapple odor, texture, and overall acceptability, respectively. A reduction of 28.5% for linalool odor demonstrated that the nanoencapsulation process improves the fresh-cut pineapple sensory characteristics compared to the free antimicrobial. However, greater acceptability was obtained for the control group (without the addition of natural antimicrobial) [31]. Higher scores for color, texture, odor, mouthfeel, and overall acceptability were obtained for maize after the nanoencapsulation of origanum EO [41]. Probably the coating material prevents the accumulation of NA in the food sample due to the controlled release profile, preventing the development of strange flavors and, therefore, improvement in general organoleptic attributes.

The addition of nanoemulsion containing cinnamon oil in sea bass fillets reduced the values of b*, increased the value of a*, and the effect on L* depended on the concentration used [47]. Despite observing variation over time of the global color in orange juice and pear juice for 16 days after adding nanoencapsulated mixture terpenes and b* attributes, a*, and L* after adding hexanal and trans 2-nanoencapsulated hexanal in apple juice, these data were compared to foods without the addition of natural antimicrobials [33,34]. The attributes related to the color of the food is crucial because it directly influences the acceptance of the food by the consumer. Increased chroma values were obtained for minced meat after nanoencapsulation of limonene, linalool, and thymol, demonstrating that they contribute to retaining the red color of the meat [43]

Only one study analyzed the rheological parameters of the food after the addition of NA. The nanoencapsulated form of cinnamon oil, an increase in hardness (12.57% to 52.27%) and reduction in cohesiveness (1.56% to 2.85%), and springiness (3.53% to 3.65%) in sea bass fillets were observed regardless of the concentration used. For adhesiveness, a reduction of 15.5% was observed for the concentration of 1429 mg/L and an increase of 6.81% in the concentration of 11,429 mg/L. When NA is added in the free form, lower hardness values can be attributed to interactions between cinnamon oil and sea bass fillet proteins, decreasing the muscle’s water-binding capacity. The hardness values decreased with the storage time for the control treatment (without NA) due to bacterial action and enzymatic autolysis. However, the samples added of NA maintained their hardness which corroborates the antibacterial activity of NA [47].

Therefore, it was observed that sensory, rheological, and composition parameters can be altered with NA, and this alteration is different when NA is nanoencapsulated. In general, nanoencapsulation prevents negative changes in the sensory characteristics of food, which is highly relevant for consumer acceptance. Further studies are needed to understand better the possible effects of free and nanoencapsulated NA on foods’ different characteristics and properties.

## 4. Conclusions and Outlook

We verified that the review’s principal method used in the nanoencapsulation of NA applied in food is emulsification. We believe that this fact is related: (i) to the mainly hydrophobic characteristic of NA, especially the EO, (ii) to the different ways (high and low energy) for obtaining emulsions and, (iii) to the fact that they demonstrate a certain ease in penetrating the bacterial cell membrane. At the end of the nanoencapsulation process, factors such as EE, particle size and homogeneous distribution, stability, and resulting charge are essential for the nanoencapsulated NA’s antimicrobial activity. They are often not analyzed in the studies.

Lipids, proteins, polysaccharides, and carbohydrates are provided to be the most common food-grade materials used as wall material in an NA nanoencapsulation system. These materials are inexpensive, non-toxic, and compatible with food formulations. However, there is still no consensus as to which wall material is best, as it will depend on the nanoencapsulation process, the core material, and the food to be inserted.

In large part, the NA does not act with a single mechanism but a set of mechanisms inhibiting and even destroying the microbial cell. Although the action of free NA can be faster, immediate, it is precisely the slow and controlled release of NA from nanostructures that makes it able to act throughout the food shelf life.

Among the analyzed microorganisms, *E. coli*, *L. monocytogenes*, *Salmonella* enterica, and *S. aureus* are frequently evaluated in food. For fruits and cereals, fungal deterioration stands out due to low pH and water content.

The NA’s efficiency, even nanoencapsulated, was different in vitro (the laboratory environment) and in situ (the food matrix). Possible interactions between NA and food constituents can be difficult in diffusion and the action against the microorganisms. Food matrices may differ in composition and characteristics, which may influence the activity of NA in different ways. Moreover, the repair of microbial cells may vary in the food compared to the simple medium.

Although nanoemulsion is promising for NA nanoencapsulation, further analysis related to the variation in the composition of foods, especially the fat content, is relevant. A study with low-fat food should also be carried out to verify whether nanoemulsions can increase the fat/lipid content in low-fat foods.

A more significant explanation of the antioxidant activity after the insertion of nNA is relevant based on the data found in this review.

Studies improving nanoencapsulation methods are relevant to quickly obtaining effective, cheap, and industrially applicable methods. Studies with direct food application are necessary since laboratory means have not shown results compatible with the food matrix’s results. A more significant explanation of the antioxidant activity after the insertion of nNA suggests that more prolonged studies in the food matrix should be attractive due to the slow and controlled release of encapsulated NA. The low-concentration combination of free and encapsulated NA in foods can be attractive due to an immediate action of free NA and a prolonged action of encapsulated NA, without affecting the sensory characteristics of food. Due to possible intermolecular interactions between the natural antimicrobials and components present in the food matrix, we believe that studies should be carried out concerning potential compounds formed from these interactions, ensuring the quality and safety of food. We agree with Lopes & Brandelli, (2018) [86] that future studies should focus more on the possible toxicological effects on human health that foods with added nanostructures may have.

## Figures and Tables

**Table 1:** Antimicrobial effect of nanoencapsulated natural antimicrobial in foods.

Encapsulation Method	Encapsulation Efficiency EE (%)	Size	Wall Material	Natural Antimicrobial	Tested Food	References
Emulsion	92.10	89.5 nm	sodium caseinate	Thyme EO	milk	[30]
Emulsion	-	10.9 nm	tween 80	Linalool	fresh cut pineapple	[31]
Emulsion	-	13 nm	sesame oil and Tween 80	Eugenol	fresh orange juice	[32]
Emulsion	-	86 and 100 nm	soy lecithin	Hexanal and trans 2-hexenal	apple juice	[33]
Emulsion	-	74.4 nm	soy lecithin	Terpenes mixture (*Melaleuca alternifólia*)	orange juice and pear juice	[34]
Emulsion	-	99 nm	peanut oil and Lecithin; Peanut oil and Sugar ester; Peanut oil and Tween 20 + monoolein	Carvacrol	zucchini (Cucurbita pepo) and cooked sausages	[35]
Emulsion	-	40 nm	PEG-40, Span 80, and sunflower oil	Oregano EO (*Origanum vulgare*)	chicken pate	[36]
Emulsion		55.5 nm	chitosan and Tween 80	Sweet orange essential oil (*Citrus sinensis*)	juice (orange and apple)	[37]
Emulsion	-	100 nm (carvacrol) and 60 nm (thyme)	triglyceride oil and Tween 80	Carvacrol and thyme oil	beef	[38]
Emulsion	-	46.7 nm	tween 80, Medium-chain triglyceride, and acetone	Trans-cinnamic acid	fresh-cut lettuce	[39]
Organogel-nanoemulsion	-	100 nm	stearic acid, sucrose stearate, peanut oil, and Tween 80	D-limonene and nisin	fresh milk	[40]
Emulsification and ionic gelation-lyophilization	88.06	32.65–52.38 nm	chitosan	*Origanum majorana* essential oil	maize	[41]
Liposome	77–87	Lysozyme and nisin 77, 80, and 86 nm	soybean PC, pectin, and polygalacturonic acid	Lysozyme and nisin	whole and skim UHT milk	[42]
Ionic gelation	-	33.76 and 54.19 nm	chitosan	Limonene, linalool, menthol and thymol	minced meat	[43]
Emulsion	-	84.7 nm	gelatin and lecithin	Thymol	milk (skim, 2% reduced-fat, and full fat) and cantaloupe juice	[44]
Vesicle (lipossomos)	94.10	81.49 nm	DOTAP and soybean PC	Bacteriocins	UHT goat milk	[45]
Emulsion	77.99	57–80 nm	chitosan and Tween 80	*Coriandrum sativum* essential oil	rice	[46]
Emulsion	-	50.71 nm	tween 80	Cinnamon oil	seabass fillets	[47]

Wall material: (PC) phosphatidyl choline; (DOTAP) (N-[1-(2,3-Dioleoiloxi) propil]-N,N,N-trimetilamônio metil-sulfato; (Span 80) sorbitan monooleate; (PEG-40) hydroxylated castor oil.

**Table 2:** In vitro efficacy of antimicrobial activity of nanoencapsulated natural antimicrobial (NA).

Natural Antimicrobial	Nanomatrix of Encapsulation	Target	NA Concentration	Control/ Comparison	Antimicrobial Effect of NA Relative to Control/Comparison	Reference
Thyme EO	Sodium caseinate-based nanoemulsion	*E. coli* O157:H7 ATCC 43895	MIC, MBC: 0.2 g/L	Free NA (MIC: 0.4 g/L; MBC: 0.6 g/L)	MIC: 50% better MBC: 50% better	[30]
		*S. aureus* ATCC 25923	MIC, MBC: 0.4 g/L	Free NA (MIC, MBC: 0.6 g/L)		
Linalool	Polysorbate 80-based nanoemulsion	*S.* Typhimurium	MIC, MBC: 1.25% (*v*/*v*)	Free NA (MIC, MBC: 6.25% (*v*/*v*); MBIC_50_: ~55%)	80% better	[31]
			MBIC_50_: ~65%		Biofilm inhibition: ~18% better	
Eugenol blended sesame oil	Polysorbate 80-based nanoemulsion	*S. aureus*	0.1% (0.003% eugenol)	Without NA	3-log reduction of bacterial population	[32]
Hexanal	Lecithin-based nanoemulsion	*S. aureus*	MIC_24h_: 2000 ppm	Free NA in 1% ethanol (2 log cfu/mL)	similar	[33]
		*L. monocytogenes*	MIC_24h_: 1000 ppm		~25% worse	
		*E. coli*	MIC_24h_: 700 ppm		~133% worse	
		*S. cerevisiae*	MIC_24h_: 800 ppm		~60% worse	
		*L. plantarum*	MIC_24h_: 400 ppm		100% worse	
*Trans*-2-hexenal	Lecithin-based nanoemulsion	*S. aureus*	MIC_24h_: 500 ppm	Free NA in 1% ethanol (2 log cfu/mL)	~67% worse	
		*L. monocytogenes*	MIC_24h_: 300 ppm		similar	
		*E. coli*	MIC_24h_: 500 ppm		similar	
		*S. cerevisiae*	MIC_24h_: 100 ppm		similar	
		*L. plantarum*	MIC_24h_: 700 ppm		~40% worse	
Terpenes-rich *Melaleuca alternifolia* EO	Lecithin-based nanoemulsion	*E. coli*	MIC: 1.0 g/L MBC: 5.0 g/L	Free NA (MIC or MBC > 5.0 g/L)	MIC > 80% better MBC: without effect	[34]
		*L. delbrueckii*	MIC: 10.0 g/L MBC: 10.0 g/L	Free NA (MIC: 5.0 g/L; MBC: 25.0 g/L)	MIC: 100% worse MBC: 60% better	
		*S. cerevisiae*	MIC: 1.0 g/L MBC: 5.0 g/L	Free NA (MIC, MBC > 10.0 g/L)	MIC > 90% better MBC > 50% better	
D-limonene	Polysorbate 20/glycerol monooleate -based nanoemulsion	*E. coli*	MIC: 5.0 g/L MBC > 25.0 g/L	Free NA (MIC, MBC > 25 g/L)	MIC > 80% better MBC: without effect	
		*L. delbrueckii*	MIC: 25.0 g/L MBC: 25.0 g/L		MIC, MBC: without effect	
		*S. cerevisiae*	MIC: 25.0 g/L MBC: 25.0 g/L		MIC, MBC: without effect	
D-limonene blended sunflower oil	Polysorbate 20/glycerol monooleate-based nanoemulsion	*E. coli*	MIC: 5.0 g/L MBC > 25.0 g/L	Free NA (MIC, MBC > 25 g/L)	MIC > 80% better MBC: without effect	
		*L. delbrueckii*	MIC: 5.0 g/L MBC > 25.0 g/L			
		*S. cerevisiae*	MIC: 5.0 g/L MBC > 25.0 g/L			
*Origanum vulgare* EO	Polyethoxylated surfactant-based nanoemulsion	*S. aureus*	MIC: 0.56 mg/mL MBC: 0.90 mg/mL	Negative control: without NA	Bacterial growth reduction: 1 log cycle	[36]
		*E. coli*	MIC: 0.60 mg/mL MBC: 3.32 mg/mL		Bacterial growth reduction: 2 log cycles	
*Citrus sinensis* EO	Chitosan nanoemulsion	*E. coli* O157:H7 *Sakai*	0.2 μL/mL	Free NA	~66% worse at pH 7 Without effect at pH 4	[37]
*Trans*-cinnamic acid	Polysorbate 80-based nanoemulsion	*S. aureus*	MIC: 0.78 mg/mL MBC: 3.13 mg/mL	Free NA	MIC, MBC: 87% better	[39]
			MBIC_50_: 0.1 mg/mL		Biofilm prevention: 74% better	
		*S.* Typhimurium	MIC: 1.56 mg/mL MBC: 3.13 mg/mL		MIC, MBC: 87% better	
			MBIC_50_: 0.2 mg/mL		Biofilm prevention: 87% better	
		*P. aeuroginosa*	MIC: 3.13 mg/mL MBC: 6.25 mg/mL		MIC: 75% better MBC: 50% better	
			MBIC_50_: 0.9 mg/mL		Biofilm prevention: 85% better	
D-limonene and Nisin	Organogel-nanoemulsion	*S. aureus*	MIC: 5.47 µg/mL	ON-D-limonene 15%	~77% better	[40]
				ON-Nisin 6%	~28% better	
		*B. subtilis*	MIC: 10.94 µg/mL	ON-D-limonene 15%	~54% better	
				ON-Nisin 6%	~27% better	
		*E. coli*	MIC: 42.15 µg/mL	ON-D-limonene 15%	~4% better	
				ON-Nisin 6%	~12,000% better	
*Origanum majorana* EO	Chitosan nanoemulsion	*Aspergillus flavus*	MIC: 1.0 μL/mL	Free NA	1.5-folds better	[41]
		Aflatoxin B_1_	MAIC: 1.0 μL/mL			
Limonene	Chitosan nanoparticle	*E. coli*	MIC:180 mg/L	Free NA	~48% better	[43]
		*S.* Typhimurium	MIC: 250 mg/L		~44% better	
Thymol		*E. coli*	MIC:200 mg/L		~55% better	
		*S.* Typhimurium	MIC:350 mg/L		~30% better	
Menthol		*E. coli*	MIC:250 mg/L		~75% better	
		*S.* Typhimurium	MIC: 375 mg/L		~66% better	
Linalool		*E. coli*	MIC: 450 mg/L		~72% better	
		*S.* Typhimurium	MIC: 500 mg/L		~72 % better	
Thymol	Gelatin-lecithin-based nanoemulsion	*L. monocytogenes*	MIC: 0.25 g/L MBC: 0.30 g/L	Free NA	similar	[44]
		*E. coli* O157:H7	MIC: 0.25 g/L MBC: 0.25 g/L		similar	
Bacteriocins (*Lactobacillus sakei*)	Liposomal nanovesicles	*L. monocytogenes*	10 µL (12.800 AU.mL^−1^ bacteriocins)	Free NA	Bacterial count reduction after 5 days at 7 °C: 5 log better	[45]
*Coriandrum sativum* EO	Chitosan nanoemulsion	AF LHP R14 strain	MIC: 0.5 μL/mL	Free NA (MIC: 0.9 μL/mL)	~44% better	[46]
		Aflatoxin B_1_	MAIC: 0.4 μL/mL	Free NA (MAIC: 0.8 μL/mL)	~50% better	

NA: natural antimicrobial; ON: organogel nanoemulsion; MIC: minimum inhibitory concentration; MBC: minimum bactericidal concentration; EO: essential oil; MAIC: minimum aflatoxin inhibitory concentration; AF LHP R14: Aflatoxigenic strain of *Aspergillus flavus*; PBS: phosphate buffered saline; MBIC_50_: minimum required to inhibit ≥ 50% biofilm formation; PIT: the phase inversion temperature method; MI: microbial inhibition.

**Table 3:** In situ antimicrobial activity of encapsulated natural antimicrobial (NA) applied in food.

Natural Antimicrobial (NA)	Food Matrix: Microorganisms Tested	Concentration In Situ	Effect Compared to Control In Situ	Mechanism of Action	References
Thyme essential oil	Milk: *Escherichia coli (EC)* and *Staphylococcus aureus* (SA)	MIC: EC: 3.5 g/L SA: 4.0 g/L MBC: EC: 3.5 g/L SA: 5.0 g/L 3.5, 5.0, 6.5 and 7.0 g/L	MIC: ↓ 12.5% for EC ↓ 50.0% for SA MBC: ↓ 12.5% for EC ↓ 35.5% for SA 3.5 g/L: ↓ 53% for EC in 25 h below the detection limit in 48 h; without effect for SA 5.0 g/L: without effect for EC ↓ 32.7% for SA 6.5 g/L: ↓ 57% for SA 7.0 g/L: ↓ 82% for SA 10 g/L: ↓ 48% in the time required to stay below the SA detection limit 11.7 g/L: ↓ 88% in the time required to stay below the SA detection limit Control: NA free	-	[30]
Linalool	Fresh cut pineapple: *S.* Typhimurium biofilms	0.3125% (*v*/*v*)	Biofilmes inhibition: ↑ efficiency Control: NA free	Damage to the membrane with the release of cytoplasmic content (proteins and acids)	[31]
Eugenol	Orange juice: Bacteria population	0.3% eugenol in the nanoemulsion	Growth of the microorganism: ↓ up to 15% Control: sodium benzoate 0.3%	Damage to the bacterial membrane	[32]
Hexanal and trans 2-hexenal	Apple juice: *Listeria monocytogenes* (LM) *Escherichia coli* (EC) *Staphylococcus aureus* (SA) *Lactobacillus plantarum* (LP) *Saccharomyces cerevisiae* (SC)	trans 2-Hexenal: 35 ppm + Hexanal: 70 ppm	Growth of the microorganism: LM: ↑ 1 log cycle for on the 2° day ↓ 1 log cycle for on the 8° day SA: ↓ 1 log cycle for on the 2° day ↓ 1.5 log cycles for on the 8° day EC: ↑ 0.5 log cycle for on the 5° day ↓ 0.5 log cycles for on the 8° day SC: ↓ 2 log cycles for on the 14° day without effect on the 22° day Control: NA free	-	[33]
Terpenes mixture (*Melaleuca alternifólia*)	Juice (orange and pear): *Lactobacillus delbrueckii*	1.0 g/L, 5.0 g/L and 10 g/L	1.0 g/L: ↓ 37.5% 5.0 g/L and 10 g/L: ↓ total Control: without NA	-	[34]
Carvacrol	Zucchini (*Cucurbita pepo*): *Escherichia coli* (EC) Cooked sausages: endogenous microbial population (EMP)	0.5 g/100g	Growth of the microorganism: ↓ 1 log cycle for EC in zucchini ↓ 1.5 log cycles for EMP in cooked meat sausage Control: without NA	-	[35]
Oregano EO (*Origanum vulgare*)	Chicken pate: *Escherichia coli* (EC) *Staphylococcus aureus* (SA)	5 g EO/100 g nanoemulsion	Growth of the microorganism: ↓ 15% for EC ↓ 11% SA Control: NA free	-	[36]
Sweet orange essential oil (*Citrus sinensis*)	Juice (orange and apple): *Escherichia coli* O157:H7 *Sakai*	0.2 μL/mL of Sweet orange essential oil	without effect Control: NA free	-	[37]
Carvacrol and thyme oil	Beef: *Escherichia coli* (EC)	8000 ppm	Growth of the microorganism: ↓ 20% to 30% for EC Control: without NA	-	[38]
trans-cinnamic acid	Fresh-cut lettuce: Aerobic mesophilic bacteria (AMB) and aerobic psychrophilic bacteria (APB)	25 mg/mL	Growth of the microorganism: ↓ 50% Control: NA free	Release of cellular constituents	[39]
D-limonene and nisin	Milk: Total microbial counts (TMC)	nisin + D-limonene 2xMIC	Growth of the microorganism: ↓ up to 80% of TMC Control: without NA	Cell membrane damage with release of cell constituents and cell lysis	[40]
*Origanum majorana* essential oil	Maize: aflatoxina B1 (AFB1)	MIC EO sem encapsular: 2.5 μL/mL MIC EO Encapsulado: 1.0 μL/mL	without effect Control: NA free	Irreversible damage to the plasma membrane with inhibition of ergosterol content, leakage of cellular ions (Ca^2+^, Mg^2+,^ and K), nucleic acids and proteins; inhibition of methylglyoxal	[41]
Lysozyme and nisin	Whole and skim UHT milk: *Listeria monocytogenes* (LM) *Salmonella Enteritidis* (SE) The mixture of strains of Listeria	1.0 mL/10 mL of milk (0.16 mg/mL nisin to 2 mg/mL lysozyme)	Growth of the microorganism: whole milk (37 °C) after 10 h: ↓ 0 to 1.5 log cycles for LM; without effect for SE skim milk (37 °C) after 10 h: ↓ 0.5 log cycle for and ↑ up to 3.5 log cycle for LM; without effect for SE whole milk (7 °C) after 25 days: ↓ 0 to 1 log cycle for LM; without effect for the mixture of strains of *Listeria* sp. skim milk (7 °C) after 25 days: ↑ 0 a 6 log cycles for LM; without effect for the mixture of strains of *Listeria* sp. Control: NA free	-	[42]
Limonene, linalool, menthol, and thymol	Minced meat: *Escherichia coli* (EC)	1000 mg/kg and 2500 mg/kg	Growth of the microorganism: Limonene: 1000 mg/kg: ↓ ~76.3% of EC 2500 mg/kg: ↓ ~79.1% of EC Menthol: 1000 mg/kg: ↓ ~66.6% of EC 2500 mg/kg: ↓ ~72.6% of EC Linanool 1000 mg/kg: ↓ ~72.1% of EC 2500 mg/kg: ↓ ~86.4% of EC Thymol: 1000 mg/kg: ↓ ~75.3% of EC 2500 mg/kg: ↓ ~83.9% of EC Control: NA free	-	[43]
Thymol	Milk (skim, 2% reduced-fat, and full fat) and cantaloupe juice: *Escherichia coli* (EC) and *Listeria monocytogenes* (LM)	1 g/L 4.5 g/L 4.5 g/L 0.6, 0.8, and 1.0 g/L 0.6 g/L	Growth of the microorganism: skim milk (48 h): ↓ 21.1% for LM ↓ 47.3% for EC 2% reduced-fat milk: ↓ 77.3% for LM in 48 h Below the detection limit for EC in 5 h full-fat milk (48 h): ↓ 19.1% for LM ↓ 72.2% for EC cantaloupe juice: ↓ >800% for LM in 24 h below the detection limit for LM and EC in 2 h, compared to 5 log cycles of the control Control: NA free	-	[44]
Bacteriocins	UHT goat milk: *Listeria monocytogenes* (LM)	12,800 AU.mL^−1^	30 °C and 7 °C: without effect Control: NA free	-	[45]
*Coriandrum sativum* essential oil (CSEO)	Rice: spore suspension	CSEO: 0.9 and 1.8 μL/mL CSEO encapsulado: 0.5 and 1.0 μL/mL	Protection against fungal infestation: MIC: ↑ 25.6% 2MIC: ↑ 21.7 AFB_1_: without effect Control: NA free	Irreversible damage to the plasma membrane with inhibition of ergosterol content, leakage of cellular ions (Ca^2+^, Mg^2+,^ and K), nucleic acids and proteins; inhibition of methylglyoxal	[46]
Cinnamon oil	Sea bass fillets: Total viable count (TVC)	1429 mg/L and 11,429 mg/L	Growth of the microorganism: 1429 mg/L: ↓ 6.9% for TVC 11429 mg/L: ↓ 7.73% for TVC Control: NA free	-	[47]

Minimum inhibitory concentration (MIC), Minimum bactericidal concentration (MBC), *Escherichia coli* (EC), Total microbial counts (TMC), Aflatoxina B1 (AFB1), Total viable count (TVC), *Coriandrum sativum* essential oil (CSEO), Endogenous microbial population (EMP), Aerobic mesophilic bacteria (AMB) and Aerobic psychrophilic bacteria (APB), *Listeria monocytogenes* (LM), *Salmonella Enteritidis* (SE), *Staphylococcus aureus* (SA), *Lactobacillus plantarum* (LP) and *Saccharomyces cerevisiae* (SC).

**Table 4:** Effects of nanoencapsulated natural antimicrobial (NA) on quality, physical and chemical properties of foods.

Natural Antimicrobial	Chemical Composition	Antioxidant Activity	Total Volatile Basic Nitrogen	Color	Lipid Oxidation	Sensory	pH and Brix	Rheology	References
Linalool	-	-	-	-	-	↑ 50% for appearance ↑ 20% for pineapple odour ↑ 14% for texture ↑ 40% for overall acceptability ↓ 28.5% for linalool odour Control: NA free	-	-	[31]
Hexanal and trans 2-hexenal	-	-	-	L*: ↑ 0.18% to 67% over the 22 days a*: ↓ 1.74% on day 0 ↓ 29% on the 19th ↑ 10% to 48% on the other days (22 days) b*: ↓ 0.57% on day 0 ↑ 0.62% to 182% on the other days (22 days) Control: without NA	-	-	-	-	[33]
Terpenes mixture (*Melaleuca alternifólia*)	-	-	-	Variation over time of the global color for 16 days: Orange juice 1.0 g/L: ↓ 1 for ΔE 5.0 g/L: ↓ 0.5 and ↑ 4.5 for ΔE 10 g/L: ↑ 2 to 14 for ΔE Pear juice 1.0 g/L: without effect 5.0 g/L: ↓ 5 and ↑ 9 for ΔE 10 g/L: ↑ 1 to 17 for ΔE Control: without NA	-	-	without effect Control: without NA	-	[34]
Oregano EO (*Origanum vulgare*)	without effect Control: NA free	-	-	-	-	-	-	-	[36]
Sweet orange essential oil (*Citrus sinensis*)	-	-	-	-	-	Orange juice ↓ 14% on the hedonic scale Apple juice: without effect Control: without NA	-	-	[37]
*Origanum majorana* essential oil	-	↓ 2.4 % in IC50 for DPPH• ↓ 9.9% in IC50 for ABTS• Control: NA free	-^+^	-	↓ ~46% for Malondialdehyde Control: NA free	Highest scores for color, texture, odor, mouthfeel, and overall acceptability Control: NA free	-	-	[41]
Limonene, linalool, menthol, and thymol	1000 mg/kg: Moisture content: without effect Total protein: without effect Ash: without effect Fat content: Linalool: ↓ 2.64% Thymol: ↓ 3.33% Menthol: without effect Limonene: without effect 2500 mg/kg: Moisture content: without effect Total protein: without effect Fat content: Menthol: ↓ 3.77% Linalool: ↑ 0.94% Thymol: ↑ 3.68% Limonene: without effect Ash: Limonene: ↑ 0.03% to 0.04% Menthol: ↑ 0.06% to 0.10% Linalool: ↑ 0.02% to 0.04% Thymol: ↑ 0.03% to 0.04% Control: NA free	Antioxidant activity (%) Limonene: ↑ ~9.5 Menthol: ↑ ~10.4 Linalool ↑ ~7.3 Thymol: ↑ ~6.0 Control: NA free	-	Changes Chroma: 1000 mg/kg: Limonene: ↑ 38.06% Linalool: ↑ 54.18% Thymol: ↑ 52.67% Menthol: ↓ 22.22% 2500mg/kg: Limonene: ↑ 0.64% Linalool: ↑ 40.7% Menthol: ↑ 1.29% Thymol: ↓ 3.4% Changes Hue: 1000 mg/kg: Limonene: ↑ 2.44% Linalool: ↑ 8.02% Thymol: ↑ 37.86% Menthol: ↑ 13.79% 2500 mg/kg: Limonene: ↑ 2.57% Linalool: ↑ 50.7% Thymol: ↑ 42.03% Menthol: ↓ 27.27% Control: NA free	Peroxide value (meq O_2_/kg fat) 1000 mg/kg: ↓ 31% to 33% 2500 kg/mg: ↓ 35% to 37.5% Control: NA free	-	without effect Control: NA free	-	[43]
*Coriandrum sativum* essential oil	-	↓ 36% in IC50 for DPPH• ↓ 26% in IC50 for ABTS•^+^ Control: NA free	-	-	↓ ~70% for Malondialdehyde Control: NA free	-	-	-	[46]
Cinnamon oil	-	-	↓ 2.5% to 15% Control: NA free	1429 mg/L: ↑ 0.37% for L* ↑ 37.8% for a* ↓ 9.01% for b* 11429 mg/L: ↓ 1.51% for L* ↑ 99.4 % for a* ↓ 48.31% for b* Control: NA free	1429 mg/L: ↑ up to 21% for TBA 11429 mg/L: ↓ up to 44% for TBA Control: NA free	-	-	1429 mg/L: ↑ 12.57% for hardness, ↓ 15.5% for adhesiveness ↓ 1.56% for cohesiveness ↓ 3.65% for springiness 11429 mg/L: ↑ 52.27% for hardness, ↑ 6.81% for adhesiveness ↓ 2.85% for cohesiveness ↓ 3.53% for springiness Control: NA free	[47]

L* (lightness), a* (redness), and b* (yellowness).
